# Some Conclusions Drawn at the Manchester Meeting

**Published:** 1920-05-22

**Authors:** 


### THE CONSEQUENCES OF DENTAL DISEASE.

### Some Conclusions Drawn at the Manchester Meeting.

In a journal which has the well-being of the public closely at heart, it is unnecessary to offer excuse for again giving space to discussion of one of the most important national problems of the day. As was announced last week, an extremely valuable exposition of the far-reaching results of dental disease in Great Britain has just been put forward in the Albert Hall, Manchester, and although among so admirable a set of papers many might well be treated individually, it will perhaps be most advantageous to summarise the whole. To the professional man, some of the facts enlarged upon may appear to be given undue prominence, but in making our abstracts we hope to bear particularly in mind those points which, though well known to the doctor himself, are certainly not realised by his patients, and are therefore just those which he must more insistently bring before them, and they be more ready to understand and accept.

### A National Defect.

It is a practically indisputable fact that we have the worst teeth of any nation. By this it is not meant that we have necessarily an unenvied monopoly in actual decay or liability of our teeth to decay. Rather must it be pointed out that we hold as a nation the highest percentage incidence over the whole population of a series of dental conditions and consequences. The chief are, irregularities of the teeth, loss of teeth due to extraction and untreated disease, unnoticed dental caries, concealed disease of the fang-tips and pyorrhœa, or disease of the tooth sockets. The great point is that some nine-tenths of the contributory causes to the country's present dental deficiences *are absolutely and readily preventable.* It is almost unnecessary to labour the secondary consequences of these conditions, but we may, apart from the more obvious results, mention defective mastication, leading through indigestion to gastric or duodenal ulcer and therefore to malignant disease. Also let us remember that dental sepsis is now universally recognised as one of the common causes of arthritis and neuritis arising primarily from toxic absorption; chronic "blood poisoning" let us call it, if that term will frighten more people into taking rational precautions in the care of their teeth. It is only by making the public understand that the campaign now opening is bringing to them something more than "an aid to fitness" that we shall get that universal and voluntary sympathy which is more than essential to success.

### Irregularities.

Let us take, therefore, these conditions in order. The first factor in preventing irregularity of the teeth is to make sure that the young infant follows as far as possible natural conditions of living. To a large extent the problem of prevention at this stage resolves itself into a question of food. This must be of such a nature and consistency, and eaten in such a manner as to maintain the mouth in a physiologically clean condition and to provide sufficient exercise of the jaws. Above all, small children should *not* be fed as though they were invalids, but should encounter a reasonable amount of coarse food such as their dental equipment is designed to cope with. The responsibility, so far, largely rests with the mother herself, and if under her care the infant develops the habit of correct mastication an invaluable start has been made. On the progress immediately following, some valuable points were put forward by Mr. A. T. Pitts. He explained that the index to the value of the teeth to any child is to be found in the amount of their "attrition" or wearing down by opposed surfaces. Marked attrition is in these days no longer found in the adult as a normal occurrence, but it is a normal sign in the child. Although the accepted immediate cause of caries is the fermentation of carbohydrate food in contact with the teeth, a variety of predisposing causes include irregularities and "crowding" of the teeth arising in a great number of cases from irregular mastication, which is itself readily shown by the "attrition marks." If there is any deficiency or perversion of the jaw movements in the child, we may expect this to be perpetuated in the adult, and the frequent occurrence of unilateral mastication unaccounted for by absence of teeth provides ample confirmatory evidence.

### Unbalanced Teeth.

It is unnecessary to enter upon the liability of such unbalanced use of the teeth in the production of food accumulation, fermentation, and ultimately caries, but it may, however, be truly said that all dental irregularities do tend, on such lines as the above, to predispose to decay. Also, in the production of this irregularity, there are factors other than deficient mastication, and as their detection depends almost entirely upon the mother's care in such matters, we would particularly have her attention drawn to the following facts, cited by several speakers.

Among the mechanical factors tending towards irregularity, we have also premature loss, or too late retention, of the milk teeth, whereby the incoming tooth is deprived of a guide or deflected from its course by an obstruction; thumb, finger or comforter sucking. Also mouth breathing may develop, where, by compelling the mouth to be kept open, deprives the upper jaw of the moulding action of the tongue. Premature *loss* of the temporary teeth is almost invariably due to extraction necessitated by *preventable* caries. Late *retention* of the temporary teeth may also be due to caries which, by causing the death of the nerve, interferes with the normal process of absorption. Hard food is required to displace a tooth when it is ready to be shed, whereas, if the food be soft, it may not exert sufficient force to dislodge it and it may be retained beyond its time.

**The Consequences of Dental Disease**—(*continued*).

Such are the simple facts, and for the moment it is best to leave the various theories concerning the effect of the growth factors, vitamines, internal secretions, etc., upon the early dentition.

Dental Caries.

Dr. James Wheatley convincingly explained that whatever the causes may be, the only way in which dental caries commences in any otherwise healthy unbroken tooth is by the solution of its enamel by acids formed from carbohydrates in the food. The enormous increase of caries during the last century has synchronised with both a great refinement in our starchy food and a great increase in our sugary food. Nowadays only a minute percentage of our population is free from carious teeth, and we believe with the speaker that this incidence has arisen from the gradual elimination from our diet of rougher foods which *cleanse* the teeth and their substitution by a host of manufactured highly refined products which allow sugary material to adhere to and ferment upon the enamel surfaces. Sweet-eating may be included if we will, but a most interesting point is raised by the fact that in a particular district under observation the teeth of those children who are, at the present moment, four to five years of age are, as compared with those of the average four- to five-year-olds of pre-war periods, far freer from active caries. These children, born about 1915, have been subjected to war restriction and modification of food practically all their lives. Their sugar consumption was halved, their flour much less "refined," their crusts eaten and not thrown away. True it is that their parents inevitably encountered a great revival of activity in health-visiting, etc., but nevertheless, the coincidence is significant.

Mention has already been made of the vital importance of mastication. The vigorous action of the tongue, the jaw muscles and the lips, and the regular working and regular disposition of the teeth in mastication all play a necessary part in a natural cleansing process. In short, the people of the country are urged, both for themselves and their children they are rearing, to realise that the appreciably poor structure of the teeth of civilised man, is largely due, directly and indirectly, to the very artificial foodstuffs which form a high proportion of his diet. The ideal is perhaps nowadays unattainable, and too urgent striving towards it results readily, indeed, in absurdity and fad; but nevertheless, we have to get back to physiological requirements to Nature's original requirements. It is said that nine-tenths of dental disease is preventable, and the statement is absolutely true. Inspection and care of the teeth themselves may do much to prevent early disease from advancing, but the essential part of the whole campaign is the education of the public, particularly the mothers of the country, in the simple physiological requirements. Fortunately, the meeting at Manchester has done much to show the way this education may be one day realised.

